# Topical Delivery of Autochthonous Lactic Acid Bacteria Using Calcium Alginate Microspheres as a Probiotic Carrier System with Enhanced Therapeutic Potential

**DOI:** 10.3390/ph19010066

**Published:** 2025-12-29

**Authors:** Sigita Jeznienė, Emilija Mikalauskienė, Aistė Jekabsone, Aušra Šipailienė

**Affiliations:** 1 Department of Food Science and Technology, Faculty of Chemical Technology, Kaunas University of Technology, Radvilėnų av. 19, 50254 Kaunas, Lithuania; 2 Institute of Pharmaceutical Technologies, Faculty of Pharmacy, Lithuanian University of Health Sciences, 50162 Kaunas, Lithuania; 3 Preclinical Research Laboratory for Medicinal Products, Institute of Cardiology, Lithuanian University of Health Sciences, 50162 Kaunas, Lithuania

**Keywords:** probiotic, encapsulation, skin cells, adhesion, topical formulation

## Abstract

**Background/Objectives:** Three distinct strains of lactic acid bacteria (LAB), isolated from naturally fermented bread sourdough and representing the local autochthonous microflora, were selected to evaluate their potential probiotic properties. In addition, we evaluated whether these strains could be used in topical formulations. **Methods:** We evaluated probiotic properties such as the ability to co-aggregate with pathogens, antimicrobial activity, inhibition of pathogenic biofilms, and ability to adhere to human keratinocyte cells. Further, bacteria were encapsulated in calcium alginate microspheres using the emulsification/external gelation method, and their viability in topical formulations was assessed. **Results:** LAB significantly inhibited biofilm formation by the tested pathogens with complete inhibition observed in certain cases. The strength and specificity of these probiotic effects varied depending on the LAB strain and the target pathogen. Furthermore, among the tested strains, *L. reuteri* 182 exhibited the highest adhesion rates, reaching 77.94 ± 1.84%. In the context of potential topical applications, the preservative present in the formulation completely inactivated the planktonic cells of *L. reuteri* 182. In contrast, encapsulation within a biopolymeric system conferred protection against the preservative’s bactericidal effect. After 35 days of storage at room temperature, viable cell counts reached 5.94 ± 0.06 lg CFU/g. **Conclusions:** Our findings confirm that local LAB strains, specifically *L. reuteri* 182 and *L. plantarum* F1, possess essential probiotic characteristics and can be effectively incorporated into preservative-containing topical formulations via efficient encapsulation strategies. This underscores the potential of these topical probiotics for skin health and highlights the need for clear regulatory guidance to ensure their safe and effective application.

## 1. Introduction

The skin is the largest organ of the human body, acting as a physical and immunological barrier to protect the body from external agents and infections. In addition to its protective function, the skin is also responsible for thermoregulatory and metabolic processes, prevents water loss, and maintains vitamin D synthesis [1,2]. It is well established that the microbiota is one of the most significant factors influencing optimal skin function. Given that skin dysbiosis is a definite contributor to skin conditions, the growing number of chronic and inflammatory skin disorders, and the increasing expense of healthcare, the manipulation of skin microbiome is paving the way for effective solutions to healthcare challenges [3,4]. The skin microbiome can be altered by various mechanisms, such as skin microbiome transplantation or skin bacteriotherapy [5]. One of the forms of bacteriotherapy is the topical application of probiotic bacteria in the form of various dermatological formulations. The oral probiotic approach is systemic, aiming to influence skin health from within by modulating the gut microbiota through the gut–skin axis. In contrast, the topical approach is local, focusing on direct interaction with the skin’s own microbial ecosystem without relying on systemic absorption or gut modulation [1,2]. These two paradigms represent fundamentally different strategies for supporting skin health. Therefore, not only are oral probiotic preparations for the treatment and prevention of diseases widely researched but also topical probiotics in skin care cosmetics for the treatment of skin diseases are increasingly used [4]. Topical probiotic preparations are considered a safe treatment option with no adverse effects, especially compared with standard therapies for skin diseases. One of the most important ways in which probiotic bacteria benefit the host is through their microbiological functionality. Probiotics must be able to compete with other pathogenic microorganisms for adhesion sites and nutrients, improve barrier function, and regulate the overall composition of the microbiome [6]. Another important probiotic characteristic is the ability to colonise the target site. Adhesion can increase the ability to interact with the host, allowing transient colonisation, prolonging their residence time to achieve the intended beneficial impact [7].

Hence, in spite of the growing number of scientific studies demonstrating the benefits of probiotic bacteria on the skin and mucosa, the use of viable probiotic bacteria in cosmetics is still complicated and poorly explored. While new products containing inactivated probiotic microorganisms, their lysates, filtrates or postbiotics have been introduced to the market, only several researchers have conducted studies to determine application potential, the mechanism of action, effectiveness, indications or contraindications and safety of viable probiotic bacteria in topical formulations [8,9]. Puebla-Barragan and Reid [10] conducted an analysis of probiotic cosmetic products, which showed that only 16% of the probiotic skin products on the market in the European Union and the United Kingdom contained live probiotic microorganisms. Although ‘postbiotics’ and ‘paraprobiotics’ and other probiotic-derived substances may have similar effects to viable probiotic bacteria, such substances may not have a beneficial long-term effect. Conversely, live probiotic bacteria, when correctly used, can confer long-term benefits to the ecosystem of human skin by living in symbiosis with the host [11].

In most cases, topical formulations contain antimicrobial preservatives. Also, to provide additional beneficial effects on the skin, various extracts and bioactive materials are used, which may influence the viability of probiotics. Consequently, in such formulations, it is difficult to maintain sufficient viability of probiotic bacteria. Therefore, in order to increase the viability of the bacteria and their resistance to adverse environmental conditions, the bacteria are encapsulated or immobilized by various methods. As the production and maintenance of a sufficient number of probiotics is still highly under-researched, our study focused not only on the probiotic properties of locally sourced bacteria, but also on their practical applicability for encapsulation and incorporation into a topical formulation. The topic of this study is of particular relevance due to the potential applications of probiotics beyond traditional dietary supplements.

The aim of this study was to assess the probiotic potential of three different LAB strains isolated from bread sourdough of local origin, reflecting the composition of the indigenous microbiota, to encapsulate viable cells within a biopolymeric delivery system and to investigate their prospective application in topical prebiotic formulations.

## 2. Results and Discussion

### 2.1. Antimicrobial Activity and Biological Competition of LAB

Adhesion to host cells is essential for the successful establishment of a bacterial pathogen infection; thus, inhibition of pathogen adhesion by antimicrobial action and/or biological competition is a significant route of action [2]. Therefore, one of the most important properties that a probiotic culture should have is an antagonistic effect on pathogens. The results of the antimicrobial effect of LAB on selected pathogenic or opportunistic pathogenic microorganisms found on human skin and mucous membrane surfaces and/or likely to cause skin or wound infections are shown in Table 1.

**Figure 1:**
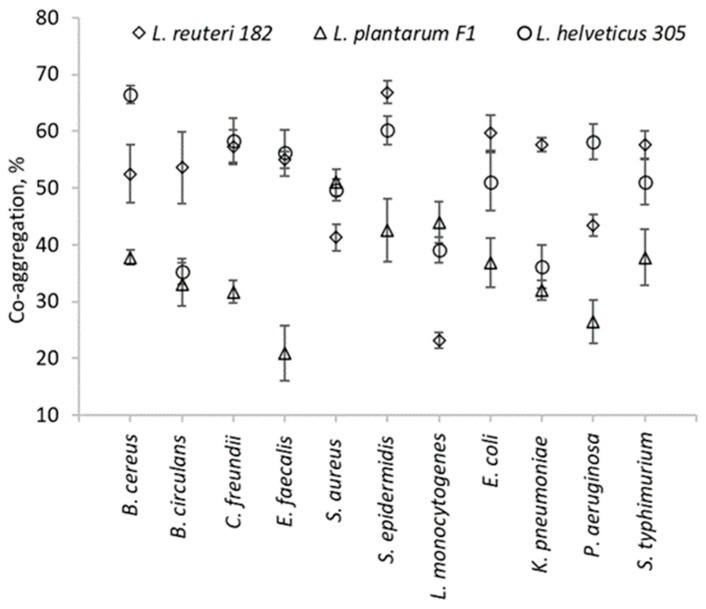
Co-aggregation ability of LAB strains with pathogens and opportunistic pathogens.

The antimicrobial effect of LAB is thought to be due to the production of small molecular compounds such as lactic acid, which are followed by a decrease in the pH of the medium, hydrogen peroxide, etc., as well as to the production of bacteriocins and bacteriocin-like compounds [12]. Our objective was to assess the overall antimicrobial activity of metabolites produced by LAB, including organic acids. As anticipated, the control MRS broth did not produce any inhibition zone. It can be seen that *L. plantarum* F1, as well as *L. helveticus* 305, exhibited antagonistic effects against all tested pathogenic cultures. In contrast, no antagonistic effect of *L. reuteri* 182 against *E. faecalis*, *S. aureus*, and *S. epidermidis* was observed. As in the case of *L. reuteri* 182, *L. helveticus* 305 showed the strongest antimicrobial activity against *C. freundii* and *L. monocytogenes*. However, comparing these two LAB strains, the effect of *L. helveticus* was statistically significantly (*p* < 0.05) stronger than that of *L. reuteri* 182. Since *L. plantarum* F1 had a similar effect on *C. freundii* and *L. monocytogenes*, it can be stated that these two mentioned cultures were the most sensitive to the antimicrobial effect of LAB metabolites. It is important to mention that Gram-negative bacteria are usually more resistant to the effect of antimicrobial substances than Gram-positive ones. This is mainly due to morphological differences in the bacterial cell wall, as the outer membrane of Gram-negative bacteria provides an additional protective layer for the cell [13]. However, after performing the correlation analysis of the experimental data, no statistical relationship between the variables was observed. Hence, it can be concluded that the antimicrobial effect of the tested LAB strains did not depend on the composition of the cell wall. The antimicrobial activity observed may result from the production of bacteriocins, which are proteinaceous substances with targeted antimicrobial properties. Additionally, organic acids produced by the strains can exert a non-specific antimicrobial effect by lowering the pH of the environment, thereby inhibiting the growth of pathogenic microorganisms. Other small molecules, such as hydrogen peroxide and diacetyl, may also contribute to the observed antimicrobial effects. However, further in-depth research is necessary to clarify the precise mechanisms responsible for the antimicrobial activity of these compounds. Another method to evaluate the antagonistic effect of LAB is the determination of co-aggregative capacity with pathogenic microorganisms. Interspecies cell–cell binding is highly specific and non-random—not all genetically different microorganisms can associate with each other [14,15]. Co-aggregation provides close interaction with pathogenic bacteria; therefore, it is a desirable property for potentially probiotic bacteria. From the presented results (Figure 1), it can be seen that each LAB co-aggregated with the pathogens very differently.

The highest co-aggregation of *L. reuteri* 182 occurred with *S. epidermidis* and reached 66.9 ± 2%. Meanwhile, the highest co-aggregation, provided by *L. helveticus* 305, was with *B. cereus* (66.5 ± 1.5%). *L. plantarum* F1 exhibited the lowest levels of co-aggregation with almost all tested strains.

**Figure 2:**
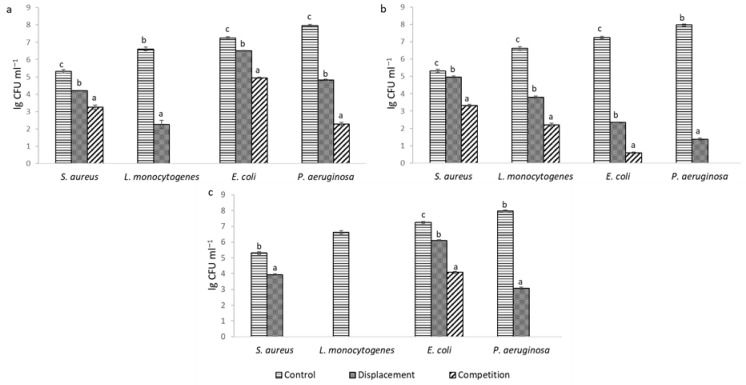
Inhibition of pathogenic biofilms: (**a**)—*L. reuteri* 182; (**b**)—*L. plantarum* F1; (**c**)—*L. helveticus* 305; results of the same pathogenic culture marked with different lowercase letters are statistically significantly (*p* < 0.05) different.

After examining the ability of LAB to inhibit pathogenic biofilms, it was found that in all cases statistically significant (*p* < 0.05) inhibition of pathogenic biofilms was achieved (Figure 2). Based on the results, it can be seen that the strength of inhibition depended on the LAB strain, the target pathogen, and the method used. *L. helveticus* 305 exhibited the highest displacement activity. Treatment with this LAB strain completely disintegrated the mature biofilm of *L. monocytogenes*.

When pathogens were co-cultivated with LAB cells, inhibition of biofilm formation was more pronounced and complete inhibition was achieved in more cases. Presumably, in the early stage of biofilm formation, LAB can interfere with pathogenic cells and inhibit their attachment more effectively. It can be noticed that the biofilm formed by *L. monocytogenes* was the most sensitive to the effect of LAB. Such results can be attributed to the strongest antimicrobial effect of LAB on this culture (Table 1). A number of authors have obtained comparable results. Lee, Lee and Paik [16] have indicated that *E. coli*, *S. aureus* and *L. monocytogenes* biofilm formation was significantly affected by the supernatant of *Lactiplantibacillus plantarum* KU200656. Barzegari et al. [17] have noted that LAB strains are a good candidate for the management of pathogenic biofilms by probiotics. Such a conclusion was also reflected in our experiment.

### 2.2. LAB Adhesion to Human Skin Keratinocytes

**Figure 3:**
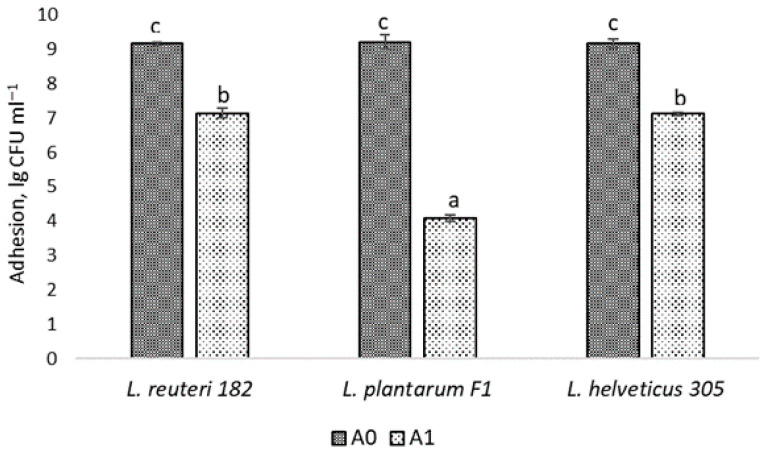
Adhesion of LAB cells to HaCaT: A0—the initial amount of microorganisms used in the experiment; A1—the number of LAB cells attached to keratinocytes; results marked with different lowercase letters are statistically significantly (*p* < 0.05) different.

In order for probiotics to have a beneficial effect, they must be able to colonize the host epidermis or epithelium, depending on the intended target site of action of the probiotic product. The epidermal layer of human skin is mainly composed of one type of cell—keratinocytes, which are in different stages of differentiation [2]. Adhesion of probiotics to host cells is a crucial step for colonization to succeed. Hence, the immortalized keratinocytes HaCaT cell line was chosen for the evaluation of adhesion to human skin and the ability of LAB to adhere to the HaCaT cells was measured. According to the results reported in Figure 3, *L. reuteri* 182 and *L. helveticus* 305 exhibited a very high adhesive capacity, which reached 77.94 ± 1.84% and 77.63 ± 2.46%, respectively (Figure 3).

The adhesion capacity of *L. plantarum* F1 to HaCaT cells, expressed as a percentage, was statistically significantly (*p* < 0.05) lower (44.23 ± 2.08%), but also relatively high compared to the results obtained by other authors. For instance, Soleimani et al. [14] investigated the probiotic formulation SYNBIO^®^, consisting of *L. paracasei* IMC and 502 *L. rhamnosus* IMC 501 and obtained 19% adhesion to keratinocytes. The authors concluded that this adhesion is sufficient for the successful use of probiotics in topical formulations. Lopes et al. [18] have examined the adhesion of probiotic cells to the keratin isolated from human skin. Among the LAB isolates investigated, the highest adhesive capacity was exhibited by *L. paracasei* L-26 (~23%). In contrast, *L. plantarum* 226v showed only ~3% adhesion.

**Figure 4:**
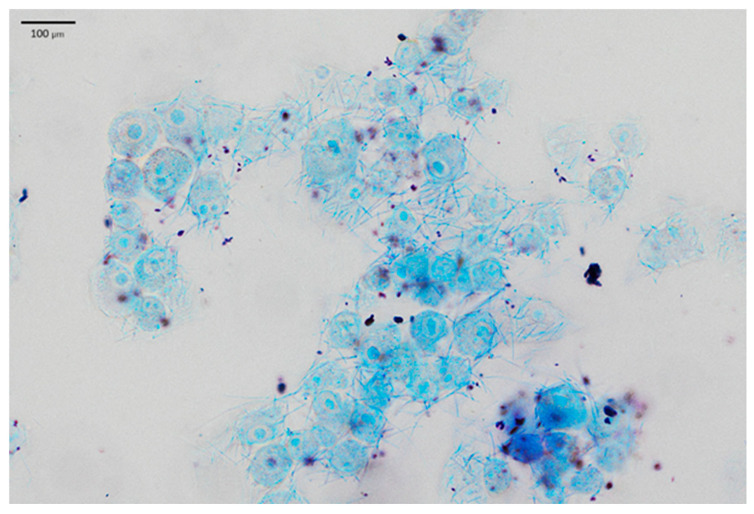
*L. helveticus* 305 bacteria adhering to HaCaT cells.

**Figure 5:**
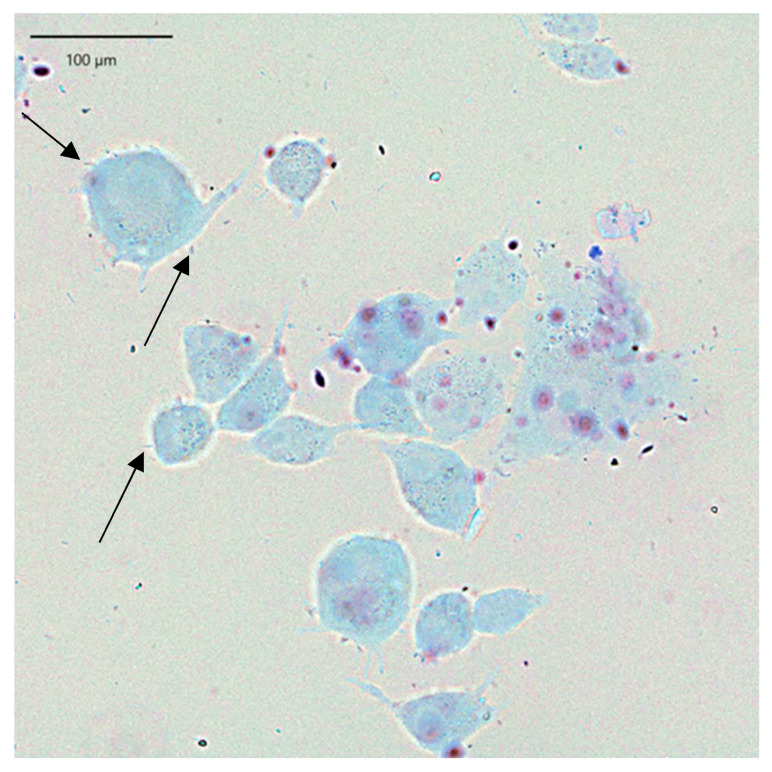
*L. plantarum* F1 bacteria (marked with arrows) adhering to HaCaT cells.

LAB adhesion was also assessed by Giemsa staining and following light microscopy, which confirmed the results obtained. In the presented Figure 4, keratinocyte cells and rod-shaped *L. helveticus* 305 bacteria abundantly surrounding them are clearly visible. On the contrary, *L. plantarum* F1, which exhibited a significantly lower capacity of adherence than *L. helveticus* 305, had a considerably lower number of bacteria attached to keratinocytes (Figure 5). It is worth emphasizing that the bacterial cell size of *L. plantarum* F1 is much smaller than that of *L. helveticus* 305, which complicated the image analysis and made it difficult to determine the number of adherent bacteria. Regarding the LAB behavior in the adhesion to keratinocyte cells, different types of biological processes and physical-chemical interactions are involved. Bacterial adhesion to human cells could be driven by hydrophobic interactions, the presence of surface binding proteins, such as fibronectin-binding proteins and surface layer proteins [7]. What is more, fimbriae or pili, which some *Lactobacillus* species can produce, may promote adhesion [19]. It is important to note that while these mechanisms are supported by literature, direct mechanistic confirmation in the context of this study would require advanced analyses such as omics approaches, surface characterization, or gene expression profiling. These techniques were beyond the scope of the present work and further in-depth investigations into the molecular basis of LAB adhesion on human skin are needed.

### 2.3. Encapsulation Efficiency and Survivability During Freeze-Drying of Microspheres with Encapsulated LAB Cells

**Figure 6:**
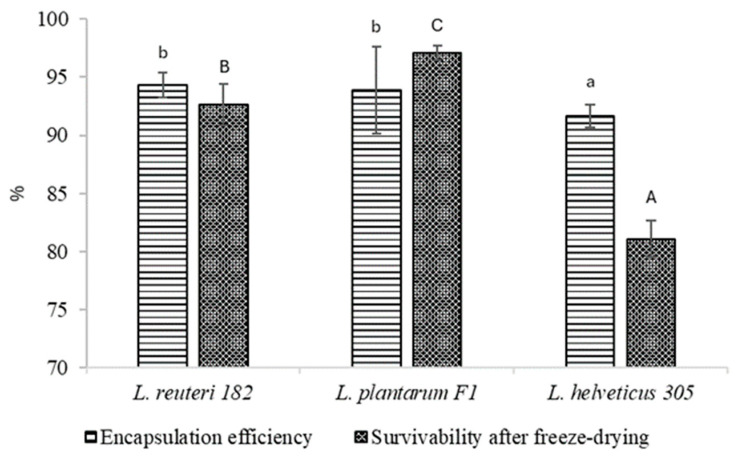
LAB encapsulation efficiency and survivability after freeze-drying. Results marked with different lowercase letters are statistically significantly (*p* < 0.05) different when comparing encapsulation efficiency; results marked with different uppercase letters are statistically significantly (*p* < 0.05) different when comparing survivability.

The encapsulation efficiency of LAB strains was analyzed, and the results are shown in Figure 6. As may be seen, in all cases the encapsulation efficiency was more than 90%. The highest encapsulation efficiency was evidenced with *L. reuteri* 182 and *L. plantarum* F1—94.35 ± 1.07% and 93.88 ± 3.73%, respectively. Significantly (*p* < 0.05), the lowest encapsulation efficiency was achieved with *L. helveticus* 305, which was 91.64 ± 0.99%.

The encapsulated LAB strains *L. reuteri* 182 and *L. plantarum* F1 withstood freeze drying and maintained the viability at 92.63 ± 1.81% and 97.05 ± 0.63%, respectively. On the contrary, unfavorable results have been achieved by other authors, who have not incorporated cryoprotectants and prebiotics into encapsulation systems. For example, Tomás et al. [20] reported a decrease in viability of *L. reuteri* CRL 1324 by 4.07 log units. The same tendencies were reflected in our previous work. Freeze-drying of calcium alginate microspheres loaded with *L. reuteri* 182 and *L. plantarum* F1, without the addition of cryoprotectants and prebiotics to the matrix, resulted in a lower survival rate than with the use of the above-mentioned substances [21]. Even with cryoprotectants used, freeze-drying of microspheres containing encapsulated *L. helveticus* 305 resulted in a loss of almost 20% of the viability of bacteria. In Miyamoto-Shinohara et al. [22] study on bacterial viability during freeze-drying, the authors observed that while survival rates were generally consistent among species of the same genus, *Lactobacillus* species showed variability potentially linked to trehalose presence. Notably, trehalose-fermenting species like *L. helveticus* exhibited low viability in comparison with non-trehalose-fermenting species *L. reuteri* and *L. animalis*, suggesting the involvement of extracellular trehalose or trehalose-like substances. Yuan et al. [23] have delivered a conclusion that the viability of probiotic bacteria is lost due to calcium damage, which increases concentration during freeze-drying. The suggested mechanism involves elevated cardiolipin production, leading to increased cell membrane fluidity and disruption of cell membrane structure [23]. Cryoprotectants are known to have a stabilizing effect on the cell membrane during freeze-drying as they may interfere with divalent cations from binding to the lipid component of the cell membrane [24]. It is quite clear that when *L. reuteri* 182 and *L. plantarum* F1-loaded microspheres were freeze-dried, the beneficial effect of the cryoprotectants was observed. Meanwhile, in-depth studies are needed to clarify why this effect was not apparent for *L. helveticus* 305 strain.

### 2.4. The Efficacy of the Preservative of the Topical Probiotic Formulation

For cosmetic products that are not classified as low microbiological risk (i.e., low water activity, high alcohol concentration, products with extreme pH, specific production conditions, etc.), a preservative efficacy confirmation test is required. Pathogenic microorganisms may be transferred to cosmetic products via contaminated raw materials, during the manufacturing process, or reasonably foreseeable use. The presence of the pathogens may lead to infection of consumers or even be crucial to immunosuppressed individuals [25,26,27]. Therefore, the efficacy of the preservative, known as the “challenge test”, must be tested in order to prevent the proliferation of pathogenic microorganisms that pose a risk to consumer health. According to the ISO 11930:2019 standard [28], it is required that the amount of vegetative bacteria decreases by at least 3-log within 7 days and does not increase until the end of the test period (28 days). The yeast count is required to decrease by at least 1-log in 7 days, and similarly, does not increase until the end of the experiment. For fungi, no increase must be observed until the end of the test period. The results presented in Table 2 indicated that the substances used for the preservation of the hand cream, 0.4% sodium benzoate and 0.2% potassium sorbate, met all the earlier mentioned requirements and provided effective protection against the growth of the pathogenic microorganisms tested.

### 2.5. Survival of Encapsulated LAB Incorporated in the Topical Probiotic Emulsion Matrix

**Figure 7:**
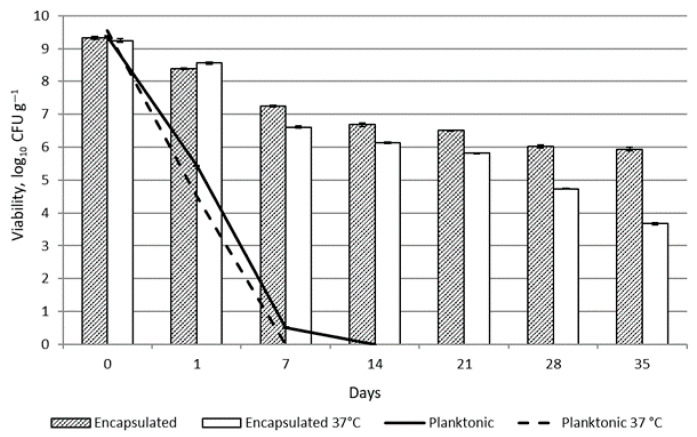
Viability of *L. reuteri* 182 in emulsion during storage.

Although *L. helveticus* 305 demonstrated significant inhibition of pathogenic biofilm formation and similar adhesion to HaCaT cells as *L. reuteri* 182, this strain displayed the lowest survivability among those tested. Meanwhile, *L. plantarum* F1 showed the lowest capacity for adherence to HaCaT cells. Therefore, based on the analysis of the probiotic properties of the LAB and the viability in microspheres, it was decided to carry out further studies on the practical application in topical formulations with *L. reuteri* 182 strain. The viability of encapsulated and planktonic *L. reuteri* 182 bacteria was investigated during storage of the emulsion at room temperature (20 °C) and under accelerated conditions at an elevated temperature of 37 °C. The results of this experiment are shown in Figure 7.

As expected, the viability of unprotected planktonic *L. reuteri* 182 cells in the topical formulation was reduced almost by half after 24 h due to the effect of the preservative in the product. After 7 days of storage at +20 °C, 0.51 ± 0.02 lg CFU g^−1^ viable bacteria were detected, whereas no viable LAB were detected at +37 °C storage temperature. By encapsulating the bacteria, the viability of *L. reuteri* 182 was preserved. After 35 days of storage at room temperature, 5.94 ± 0.06 lg CFU g^−1^ of viable bacteria were detected. Under accelerated conditions, the viable bacterial count was lower and reached 3.67 ± 0.03 lg CFU g^−1^. The development of formulations with live microorganisms is fundamentally different from the formulation of small-molecule active-containing products intended for topical application. Carbol, Osborne, Tan and Varma [29] addressed the strategy of preserving the formulation appropriately but using a sufficiently narrow-spectrum preservative to preserve the viability and/or efficacy of the probiotic active ingredient. the authors concluded that it is possible to formulate a preserved topical product containing living microorganisms. Similar results have been obtained by Łętocha et al. [8]. The results showed that when *Lactobacillus casei* cells were added to emulsion formulation containing preservatives in free form, no viable bacteria were detected at the end of the experiment (after 120 days). On the contrary, the viability of the probiotic encapsulated in alginate microspheres was 6.13 lg CFU g^−1^ after 120 days of storage [8]. However, there is a lack of scientific studies investigating the survival of live probiotic bacteria in cosmetic matrices in order to be able to comprehensively compare the results obtained. An additional limitation that should be noted concerns regulatory frameworks. EU regulations pose significant challenges for the cosmetics industry, as current legislation does not allow the use of live probiotic bacteria in cosmetic products at high levels. At present, EU cosmetic law does not distinguish between pathogenic and beneficial bacteria, and a single, general regulatory framework applies to all microorganisms. Cosmetic products marketed within the European Union are required to maintain microbiological stability and safety throughout their entire shelf life. The detection of any viable microorganisms is regarded as a potential risk to consumer health. Consequently, manufacturers are required to utilize alternatives such as postbiotics or fermented extracts, despite the fact that these substitutes may not replicate the full spectrum of biological benefits provided by live probiotics.

## 3. Materials and Methods

### 3.1. Determination of Probiotic Properties of Lactic Acid Bacteria

#### 3.1.1. Microorganism Growth Conditions

Lactic acid bacteria (LAB) *Lactiplantibacillus plantarum* F1, *Lactobacillus reuteri* 182, and *Lactobacillus helveticus* 305 were selected for research, which were isolated from spontaneous bread sourdough and obtained from the microbiology laboratory of the Food Institute of Kaunas University of Technology (Kaunas, Lithuania). Pure cultures were maintained on MRS Agar (De Man–Rogosa–Sharpe) (Biolife, Monza, Italy) at +1 °C. *L. plantarum* F1 and *L. reuteri* 182 were maintained under normal atmosphere, and *L. helveticus* 305 under anaerobic conditions (<1% O_2_; 9–13% CO_2_) in sealed Oxoid AnaeroJar containers (Thermo Fisher, Oxford, UK) with CO_2_ gas generating oxygen absorber AnaeroGen (Thermo Fisher, Oxford, UK). Other microorganisms used for research and their cultivation conditions are given in Table 3.

#### 3.1.2. Co-Aggregation Assay

Determination of LAB co-aggregation with pathogens was performed according to Tuo, Yu, Ai, Wu, Guo, and Chen [30] with several modifications. The LAB and pathogen cells were harvested by centrifugation at 4427× *g* for 10 min at 4 °C temperature. Supernatant was discarded, the cell pellet was washed twice and resuspended in the phosphate-buffered saline (PBS). The optical density at a wavelength of 600 nm (OD_600_) of LAB and tested pathogenic cells suspensions (Table 3) were adjusted to 0.25 ± 0.05 and were mixed (2 mL each) by shaking the test tubes with a vortex mixer and incubated at 37 °C temperature without agitation. The OD_600_ of carefully pipetted upper layers of LAB-pathogen mixed suspensions was measured after 24 h. In order to minimize the influence of simple sedimentation or gravitational settling, the OD_600_ of individual strains was assessed under the same conditions. Co-aggregation (%) was calculated according to Equation (1) and expressed as a percentage.

(1) $$Co\text{-}aggregation=\left(\left(\frac{A_{pat}+A_{probio}}{2}-A_{mix}\right)/\left(\frac{A_{pat}+A_{probio}}{2}\right)\right)\times 100\%,$$

where here, *A_pat_* and *A_probio_*—pathogen and LAB cell suspensions OD_600_, respectively, before mixing (at the initial moment); *A_mix_*—suspension mixture of pathogens and probiotics OD_600_ after 24 h.

#### 3.1.3. Evaluation of the Antimicrobial Activity of LAB Metabolites

The antagonistic effect of LAB metabolites on selected microorganisms (Table 3) was determined by the agar diffusion method. Cell suspensions of activated pathogenic microorganisms were prepared by dilution with sterile PBS. Concentrations were adjusted using a McFarland 0.5 standard (Liofilchem, Italy) to a final suspension density of ~10^8^ CFU mL^−1^. 100 µL of the prepared suspension was mixed with 12–15 mL of PCA (Plate Count Agar, Liofilchem, Italy) at +45 °C, poured into Petri dishes and left to solidify on a horizontal surface. Then, the wells with a diameter of 6 mm were cut out with a sterile metal cylinder from which the nutrient medium was removed. 50 µL of 24 h incubated MRS (De Man, Rogosa, Sharpe) Broth medium (Biolife, Italy) with LAB cells was poured into the wells. As controls, sterile MRS broth was used. Prepared samples were incubated at 37 °C for 24–48 h. The antimicrobial effect was evaluated by measuring the clear zone formed around the wells in three directions (mm) and calculating the average (including the diameter of the well). Inhibition was considered significant if the mean diameter of the zone of inhibition was 7.5 mm or greater.

#### 3.1.4. Inhibition of Pathogenic Biofilm Assay

The ability of LAB strains to inhibit pathogenic biofilms has been determined by competition and displacement methods by Woo and Ahn [31] with some modifications. The pathogenic microorganisms selected for the study were *E. coli*, *L. monocytogenes*, *P. aeruginosa*, and *S. aureus*, all of which were capable of forming biofilms. 200 μL of BHIB (Brain Heart Infusion Broth, Liofilchem, Italy) medium inoculated with an activated pathogenic culture was dispensed into the wells of sterile microplates with a U-type bottom and incubated under optimal conditions for 48 h. After incubation, wells were washed twice with sterile PBS to remove planktonic cells. The resulting biofilms were used for the displacement test and as a control sample. When inhibition was tested by the displacement method, 100 μL of LAB cell suspension was added to the wells (~10^7^ CFU/well) of the microplates containing the pathogenic biofilm and incubated for 24 h at +37 °C. The microplates were then washed twice with sterile PBS, the biofilms were disrupted with a sterile microbial loop, and the cells were collected. The number of colonies forming units of pathogenic bacteria was calculated by the pour-plate technique using appropriate selective medium (Table 3). When biofilm inhibition was determined by the competition method, pathogenic and LAB strains were co-cultivated in microplates for 48 h at a temperature of +37 °C. The wells were washed, the biofilm was disrupted using a microbiological loop, and the number of colony-forming units of pathogenic bacteria was calculated.

#### 3.1.5. LAB Adhesion to Epidermal Cells

The ability of LAB to adhere to human skin cells was assessed by measuring adhesion to keratinocytes. A commercial immortalized keratinocyte (HaCaT, RRID:CVCL_0038, CLS Cell Lines Service GmbH, Eppelheim, Germany) cell line was cultured in DMEM medium (Dulbecco’s modified Eagle’s medium, Gibco, ThermoFisher Scientific, Waltham, MA, USA) with 10% FBS (fetal bovine serum, Gibco, ThermoFisher Scientific, Waltham, MA, USA) supplemented with a 1% mixture of penicillin and streptomycin antibiotics (Pen-Strep, Gibco, Thermo Fisher Scientific, Waltham, MA, USA). HaCaT cells were grown in 24-well plates at 37 °C in incubators maintained at 5% CO_2_ until 80–85% confluency was achieved. The old nutrient medium was then discarded, and the HaCaT cells were washed with PBS to remove residual medium, and 1 mL of the test LAB suspension (10^9^ CFU mL^−1^) was transferred to the wells and incubated for 1 h at a temperature of 37 °C. After incubation, cells were detached by exposure to 0.1% Triton X-100 (Sigma-Aldrich Chemie, Schnelldorf, Germany) solution for 10 min. Collected cells were washed and resuspended in sterile saline. Then, appropriate dilutions were plated in MRS nutrient medium and incubated under optimal conditions. After 24–48 h, colony forming units were counted and adhesion was calculated as a percentage according to Equation (2) [30]:

(2) $$Adhesion=\left(\frac{{Log}_{10}A_{1}}{{Log}_{10}A_{0}}\right)\times 100\%,$$

where *A*_0_—LAB cells, initially added to each well, CFU mL^−1^; *A*_1_—number of adherent LAB cells, CFU mL^−1^.

Additionally, the adhesion of LAB to HaCaT cells was tested microscopically according to Coman et al. [32] with several modifications. 250,000 HaCaT cells were plated in a 12-well plate with coverslips at the bottom and incubated for 24 h under optimal conditions. Then, HaCaT cells were washed twice with PBS, 1 mL of LAB suspension was transferred to the wells and incubated for 1 h at a temperature of +37 °C. To remove non-adherent LAB cells, the wells were washed three times with sterile PBS and the remaining cells were fixed with 4% paraformaldehyde solution for 10 min. The wells were then washed with PBS and filled with 400 µL of Giemsa dye solution (Sigma-Aldrich Chemie, Germany). The dye was left on for 15 min, the wells were washed three times with PBS and air-dried at room temperature. The coverslips were then removed from the wells, and antifade mounting medium (Vectashield, Vector Laboratories, Newark, CA, USA) was placed on microscope slides. The slides were observed and analyzed using an Apexview APX 100 microscope (Olympus, Tokyo, Japan) equipped with an OCRA-fusion C14440 digital camera (Hamamatsu Photonics, Hamamatsu City, Japan) in the CellSens software (Olympus, Tokyo, Japan) environment (Olympus, Tokyo, Japan).

### 3.2. LAB Encapsulation and Application in Topical Formulation Preparation

#### 3.2.1. Microencapsulation Procedure

Based on our previous research in which we investigated the different encapsulation systems, it was decided to use the emulsification/external gelation method for the preparation of microspheres [21] with following components. The encapsulation matrix was prepared by dissolving 2% of sodium alginate, 2% inulin, and 4% trehalose in water and was left overnight for complete rehydration. Prepared sodium alginate and prebiotics solution was mixed with LAB cells suspension (10^9^ CFU mL^−1^). The combined suspension was slowly poured into sterile sunflower oil in a mass ratio of 1:3, which was mixed with a magnetic stirrer at a constant 400 rpm. min^−1^ speed. To achieve a smaller size of dispersed droplets, the oil phase contained 0.85% Tween 80. The emulsion was mixed at the same speed for another 30 min and 0.1 M Ca-lactate solution was added to the resulting emulsion, causing the formation of cross-linked calcium alginate microspheres. The microspheres were washed with sterile PBS three times and collected by centrifugation at 4 °C 4427× *g* for 10 min. Microspheres were freeze-dried and stored at 4 °C in an airtight container.

#### 3.2.2. Enumeration of the Encapsulated Bacteria and Survival Assay

To determine the encapsulation efficiency, viable cells per gram of calcium alginate microspheres were counted. In order to disrupt the microspheres and to release encapsulated bacteria, the first 10-fold dilution was made in 2% sterile sodium citrate solution. The encapsulation efficiency E (%) of LAB cells was calculated according to Equation (3):

(3) $$E=\left(\frac{{Log}_{10}N}{{Log}_{10}N_{0}}\right)\times 100\%$$

where *N*_0_—LAB cells, initially added to encapsulation matrix, CFU mL^−1^; *N*—number of viable LAB cells in microspheres, CFU g^−1^.

To evaluate the survival of the bacteria during freeze-drying, freshly prepared (wet) microspheres and freeze-dried microspheres were disrupted in sodium citrate and the number of colony-forming units per gram of microspheres was calculated. Survival rate was expressed as a percentage according to Equation (4):

(4) $$Survival=\left(\frac{{Log}_{10}S}{{Log}_{10}S_{0}}\right)\times 100\%$$

where *S*_0_—number of viable LAB cells in wet microspheres, CFU g^−1^; *S*—number of viable LAB cells in freeze-dried microspheres, CFU g^−1^.

#### 3.2.3. Topical Prebiotic Formulation Preparation

In order to create a topical formulation that would be suitable for the care of the skin on the hands or body, while mimicking the specific conditions in the product, an emulsifier system has been chosen and active ingredients beneficial to human skin have been added to it. The selected composition of topical emulsion is presented in Table 4.

First, the ingredients of phases A and B were heated in a water bath until a temperature of +75 ± 2 °C. While stirring with a magnetic stirrer at 500 rpm min^−1^ speed, the phase B ingredients were poured into aqueous phase A to form a primary emulsion. Next, the emulsion was homogenized for 5 min at 9000 rpm. min^−1^ speed. The obtained emulsion was transferred to be mixed with a vertical mixing mixer Ika Eurostar 40 Digital (Ika, Straufen, Germany) with a stainless steel “propeller”-shaped nozzle at 400 rpm·min^−1^ speed. After the emulsion had cooled to a temperature of +40 ± 2 °C, the ingredients of phase C were added. Mixing at the specified parameters continued until the ingredients were evenly distributed. The pH of the emulsion was adjusted to pH = 5.0 ± 0.25 using a 50% lactic acid solution (phase D) and the mixture of phase E ingredients was added. The prepared hand cream was stored in closed containers at room temperature.

#### 3.2.4. Preservative Challenge Test

The effectiveness of the preservative system was determined based on the EN ISO 11930:2019 standard [28]. The test samples were infected with 0.1 mL suspensions of 10^5^–10^6^ CFU mL^−1^ of *E. coli*, *S. aureus*, *P. aeruginosa*, and 10^4^–10^5^ CFU mL^−1^ of *Candida albicans* and *Aspergillus niger* pathogens. 10 g of the test sample was taken for each pathogenic microorganism. Samples with bacteria and yeast were incubated at a temperature of +32.5 ± 2.5 °C, and with *A. niger* at a temperature of +22.5 ± 2.5 °C. The samples were monitored for a total of 28 days, with an evaluation of logarithmic decrease in the CFU g^−1^ of viable pathogenic microorganisms compared to the baseline value at 7-day intervals. The first 10-fold dilution was carried out using D/E Neutralizing Broth (Liofilchem, Italy) medium. The data obtained are compared with the parameters presented in Table 5.

If the parameters of the product meet the requirements of criterion A, the product is considered to be protected against the proliferation of microorganisms that are potentially harmful to the user. Compliance with the requirements of criterion B indicates that the product can be considered safe for the user only if additional protective measures are applied. When the parameters of the product do not satisfy any of the criteria, such a cosmetic product cannot be supplied to the market.

#### 3.2.5. Determination of LAB Viability in Topical Formulation Products Under Accelerated Conditions

10 mL of non-encapsulated LAB suspension or 10 g of microspheres were mixed with 100 g of the tested cosmetic product and stored for 5 weeks in transparent PET plastic containers with screw caps at +37 °C. Samples were analyzed after 1, 7, 14, 21, 28, and 35 days. LAB viability was determined according to the methodology described in Section 3.2.2. In order to avoid false negative results, after disrupting the capsules in sodium citrate, a second 10-fold dilution was performed using the liquid nutrient medium D/E Neutralizing Broth, thus neutralizing the antimicrobial effect of the preservatives used in the product.

### 3.3. Statistical Analysis

Data were expressed as the means ± standard deviation. All the experiments were conducted in triplicate and data were subjected to one-way analysis of variance (ANOVA) followed by the Fisher LSD test using Statistica 10.0 software. A *p*-value < 0.05 was assumed statistically significant.

## 4. Conclusions

This study establishes the scientific basis for assessing the potential of lactic acid bacteria of local food origin for use in topical probiotic formulations. In summary, the results of this experiment demonstrated the probiotic properties of LAB isolates collected from locally sourced bread sourdough and their ability to compete with pathogenic microorganisms. Moreover, the tested strains *L. reuteri* 182 and *L. plantarum* F1 demonstrated a pronounced ability to adhere to epidermal keratinocytes, indicating their potential to transiently colonize human skin. These strains were encapsulated in calcium alginate microspheres, and the addition of trehalose and inulin was found to effectively enhance bacterial viability during freeze-drying. Such bacterial shielding has enabled the use of viable probiotics in topical formulations, even though the efficacy of the preservative used in the topical formulation has been confirmed. It is important to note that topical probiotic products require a viable microorganism count of 6–8 lg CFU g^−1^ to be effective. The results of this study demonstrate that the encapsulation system can maintain this level of viable bacteria even in preserved products, offering real promise for the successful application of live bacteria in preserved topical formulations that serve as cosmeceuticals. As scientists further investigate the impact of the microbiome on the skin and the intriguing gut–skin axis, new research will help to shed light on the role of topical probiotics of local origin in the treatment of various skin diseases. However, the study also underscores the need for further research to elucidate the mechanisms of action, long-term safety, and efficacy of such products, as well as the importance of establishing clear regulatory guidelines to ensure their safe and effective use.

## Figures and Tables

**Table 1:** Antimicrobial activity of LAB against pathogenic and opportunistic pathogenic microorganisms.

Microorganisms	Diameter of Inhibition Zone, mm		
	*L. reuteri* 182	*L. plantarum* F1	*L. helveticus* 305
**Gram-Positive**			
*B. cereus*	11.3 ± 1.0 ^aA^	17.8 ± 0.5 ^bB^	18.0 ± 0 ^cB^
*B. circulans*	13.0 ± 0.4 ^bA^	19.4 ± 0.5 ^cB^	19.3 ± 0.5 ^dB^
*C. freundii*	19.1 ± 1.0 ^cA^	19.3 ± 0.5 ^cA^	21.3 ± 0.5 ^eB^
*E. faecalis*	no zone	14.5 ± 0.6 ^aA^	16.4 ± 0.5 ^bB^
*S. aureus*	no zone	16.8 ± 0.5 ^bB^	14.5 ± 0.6 ^aA^
*S. epidermidis*	no zone	15.5 ± 0.6 ^aA^	16.5 ± 0.6 ^bB^
*L. monocytogenes*	19.5 ± 0.6 ^cA^	19.6 ± 0.5 ^cdA^	21.3 ± 0.5 ^eB^
**Gram-Negative**			
*E. coli*	11.3 ± 0.5 ^aA^	15.5 ± 0.6 ^aC^	13.5 ± 0.6 ^aB^
*K. pneumoniae*	11.1 ± 0.3 ^aA^	15.0 ± 0 ^aB^	16.0 ± 0.8 ^bC^
*P. aeruginosa*	14.5 ± 0.6 ^dA^	20.6 ± 0.5 ^dC^	18.3 ± 0.5 ^cB^
*S. typhimurium*	11.5 ± 0.6 ^aA^	17.0 ± 0.8 ^bB^	16.8 ± 0.5 ^bB^

The results are presented as means with standard deviations; different lowercase letters in columns and different uppercase letters in rows indicate statistically significant (*p* <0.05) differences.

**Table 2:** Hand cream preservative system efficacy test results.

Microorganism	Logarithmic Reduction in Time T_x days_			Statement of Conformity
	T_7_	T_14_	T_28_	
*E. coli*	4.37	5.32	5.32	comply with criteria A
*S. aureus*	5.17	5.17	5.17	comply with criteria A
*P. aeruginosa*	4.47	5.61	5.61	comply with criteria A
*C. albicans*	3.28	4.72	4.72	comply with criteria A
*A. niger*	-	3.63	3.63	comply with criteria A

**Table 3:** Microorganisms and cultivation conditions.

Microorganism	Growth Media	Selective/Differential Media
*Bacillus cereus*	Nutrient Broth (NB, Liofilchem, Roseto degli Abruzzi, Italy)	-
*Bacillus circulans*	NB	-
*Citrobacter freundii*	NB	-
*Enterococcus faecalis*	NB	-
*Escherichia coli*	Brain Heart Infusion Broth (BHIB, Liofilchem, Roseto degli Abruzzi, Italy)	Eosin Methylene Blue Agar (Oxoid, Hampshire, UK)
*Klebsiella pneumoniae*	NB	-
*Listeria monocytogenes*	BHIB	Agar Listeria Acc. to Ottaviani and Agosti (Biolife, Monza, Italy)
*Pseudomonas aeruginosa*	BHIB	Pseudomonas Agar Base (Oxoid, Hampshire, UK)
*Salmonella typhimurium*	BHIB	-
*Staphylococcus aureus*	NB	Bair Parker Agar (Liofilchem, Roseto degli Abruzzi, Italy)
*Staphylococcus epidermidis*	NB	-

**Table 4:** Composition of hand cream.

Phase	INCI Name	Concentration, %
A	Aqua	q.s. to 100
	*Aloe barbadensis* leaf juice powder	0.1
	Glycerin	4.0
	Sodium hyaluronate	0.1
B	*Simmondsia chinensis* seed oil	10.0
	*Helianthus annuus* seed oil	20.0
	Cetearyl olivate	1.9
	Sorbitan olivate	1.9
	Glyceryl stearate	1.9
C	Tocopherol	1.0
	Glycerin (and) Aqua (and) *Tassmannia lanceolata* fruit/leaf extract	2.0
	Calcium alginate microspheres	10.0
	Parfum	1.5
D	Aqua (and) Lactic acid	0.1
E	Aqua	2.0
	Sodium benzoate	0.4
	Potassium sorbate	0.2

**Table 5:** Limits for the validation of preservative efficacy.

Microorganism	Criterion	Logarithmic Decrease		
		7 d	14 d	28 d
Bacteria	A	≥3	≥3	≥3
	B	-	≥3	≥3
*C. albicans*	A	≥1	≥1	≥1
	B	-	≥1	≥1
*A. niger*	A	-	≥0	≥1
	B	-	≥0	≥0 ^a^

^a^—no increase in counts from the previous interval.

## Data Availability

The original contributions presented in this study are included in the article material. Further inquiries can be directed to the corresponding authors.
